# Supplementary material for: Life at high temperature observed in vitro upon laser heating of gold nanoparticles
Source: Nat Commun. 2022 Sep 12;13:5342. doi: 10.1038/s41467-022-33074-6 (PMC9468142; doi:10.1038/s41467-022-33074-6)
Supplement: Supplementary file 1 — Supplementary information file [file 41467_2022_33074_MOESM1_ESM.pdf]

SUPPLEMENTARY INFORMATION

Life at high temperature observed in vitro upon laser heating of gold nanoparticles

Céline Molinaro,[1,*] Maëlle Bénéfice,[1,*] Aurore Gorlas,[2] Violette Da Cunha,[2] Hadrien M. L. Robert,[1] Ryan Catchpole,[2] Laurent Gallais,[1] Patrick Forterre,[2] Guillaume Baffou[1,†]

[1] Institut Fresnel, CNRS, Aix Marseille University, Centrale Marseille, 13013 Marseille, France

[2] Université Paris-Saclay, CEA, CNRS, Institute for Integrative Biology of the Cell (I2BC), 91198, Gif-sur-Yvette, France

* *These authors contributed equally*

† *Corresponding author: guillaume.baffou@fresnel.fr*

1. Fabrication of the drilled coverslip

This top coverslip was fabricated by a glass laser cutting technique. The laser processing system is based on a commercial femtosecond-diode-pumped ytterbium amplified laser source (S-Pulse HP, Amplitude Systemes) operating at 1030 nm (FWHM 5 nm) with a spatially Gaussian beam profile. The source is coupled in a dual-axis scanning galvometric system (GVS012/M, Thorlabs) with metallic mirrors and a 100 mm focal length f-theta lens (FTH100-1064, Thorlabs). The 150 µm thickness coverslips were processed with the following parameters: beam diameter 60 µm, repetition rate 1 kHz, 0.5mJ energy per pulse, 10µm steps.

2. Influence of the coverslip's hole on growth of bacteria

A hole was drilled on the top coverlip to enable as exchange (in particular oxygen) with the surroundings and avoid bacteria asphyxia. The sample (as in Figure 1b) has been incubated for an hour at 60°C, and then imaged using phase-contrast to evidence the effect of the hole on the bacterial growth. As observed in Fig. S1, far under the coverslip (above 1 mm from the

hole edge), no growth is observed. However, closer to the hole, effective growth is achieved, despite the spatial confinement imposed by the coverslip.

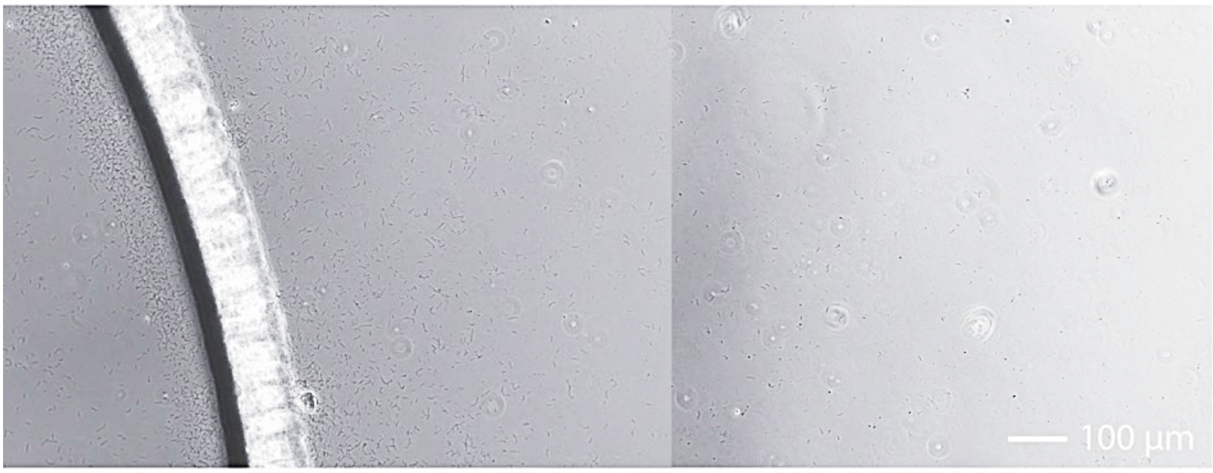

Figure S1: Phase-contrast images after 1 h of incubation at 60°C using the sample design for the laser heating stage (as in Fig. 1b). The coverslip hole is located on the left side. Its boundary is the vertical big line on the left of the image. Two images of stitched to observe that the growth is limited after a few 100s of micrometers. After around 1.1 mm underneath the coverslip from the side of the hole, no growth is observed. This phenomenon was observed each time the experiments were conducted, that is at least 20 times.

Experiments were also conducted with a plain coverslip (no hole), to confirm that cells cannot grow when confined, even with local heating. The sample was not pre-incubated, according to our previous study with this bacteria,[1] to prevent any sporulation from the whole sample. The laser beam used in this experiment was uniform and circular (no SLM). Experiments were performed 6 times, over various areas during a day. The laser was turned on during at least 1h30 for each experiment. No growth has been observed anywhere (see an example in Figure S2). When this experiment was performed at the vicinity of the hole, growth was observed around 45 min after the laser was turned on (or even after a few minutes if the experiment was preceded by a 1h-long pre-incubation).

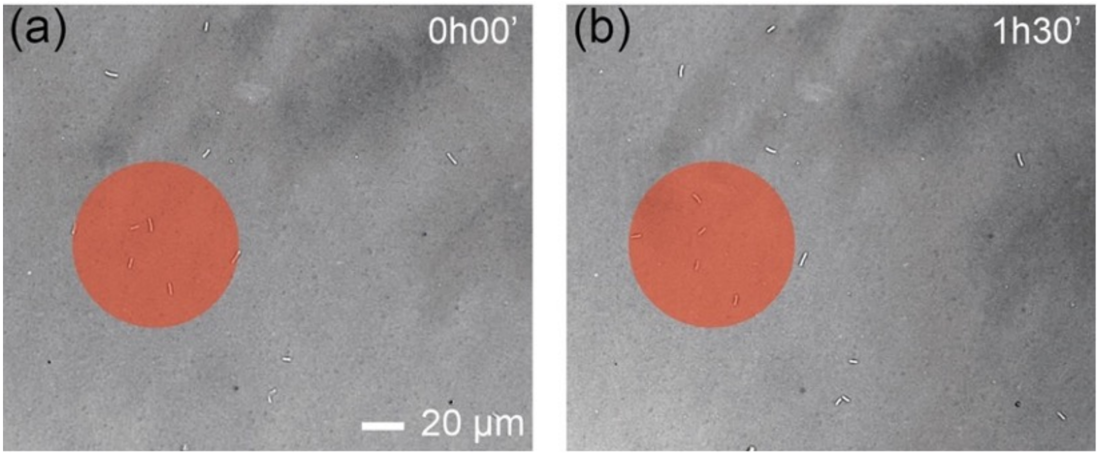

Figure S2: Images of *G. stearothermophilus* just before **(a)** and after 1h30 of gold NPs laser heating **(b)**, performed with a plain top coverslip (no hole). No growth was observed upon laser heating. The red disc indicates the laser spot. The scale bar is the same on the two images. Experiment conducted once.

3. Effective activation of thermophiles without spatial light modulation

Figures S3 and S4 evidence the effective activation of thermophiles without SLM-assisted laser shaping.

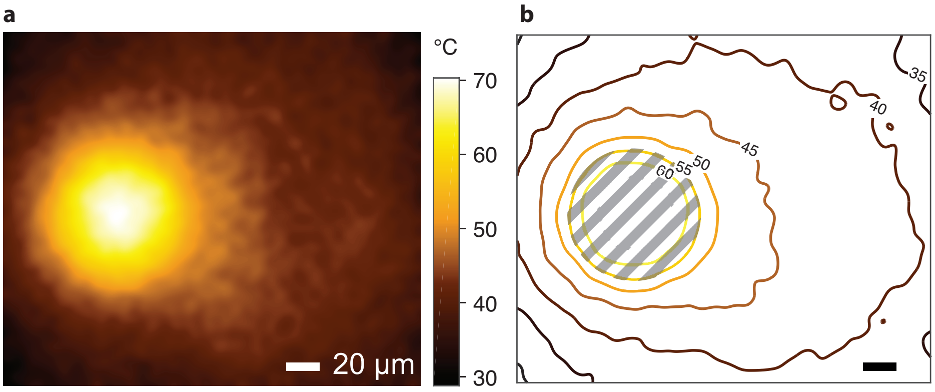

Figure S3: **(a)** Temperature map measured by QPM for a uniform laser beam, creating a Gaussian-like temperature profile. **(b)** Associated isotherms. The laser beam size is represented by the grey hatched area. Experiment conducted once.

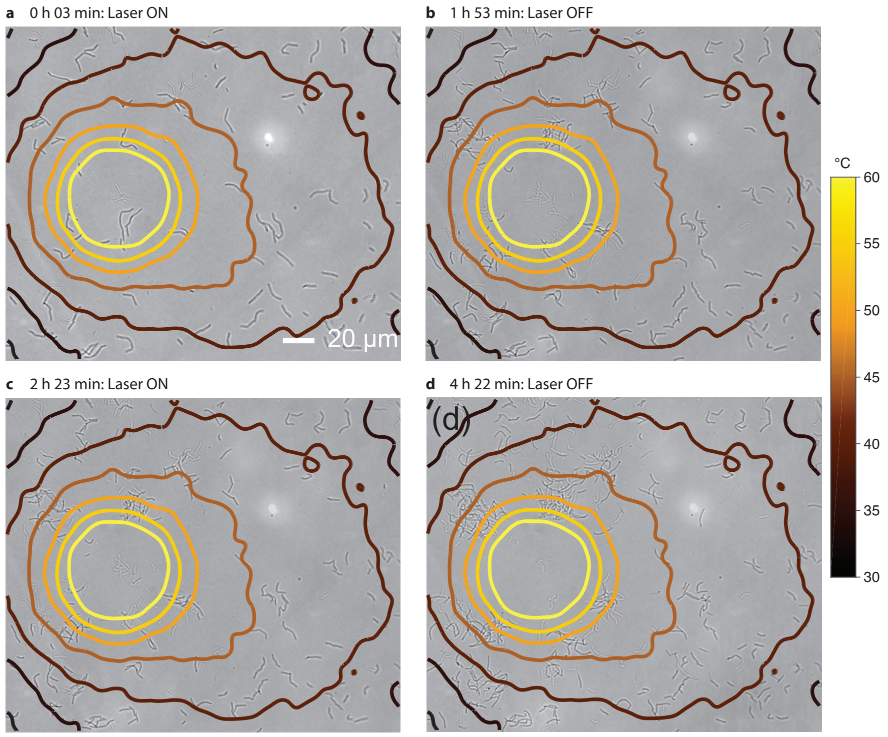

Figure S4: Images of the growth of the bacteria through laser heating at different time, extracted from Movie M1, superimposed with measured isotherms. The laser was switched on t=3 min **(a)**, then switched off at 1h53' **(b)** and switched on again at 2h23' **(c)** for 2 hours **(d)**. The laser beam intensity was uniform over a circular area (the SLM was not used) so that the temperature distribution was Gaussian.

4. The Cardinal Model

Fitting of the growth rate as a function of temperature was done using the Cardinal Model depicted by Kakagianni *et al.* in Ref.[2]. The fitting function $G(T) = G_0 g(T)$ where $g(T)$ reads

$$g(T) = \frac{(T - T\mathrm{max})(T - T\mathrm{min})^2}{(T\mathrm{opt} - T\mathrm{min})[(T\mathrm{opt} - T\mathrm{min})(T - T\mathrm{opt}) - (T\mathrm{opt} - T\mathrm{max})(T\mathrm{opt} + T\mathrm{min} - 2T)]}$$

and where G_0 is the maximum growth rate, reached at T_{opt}. The three temperature fitting parameters correspond to what is represented in Fig. S5:

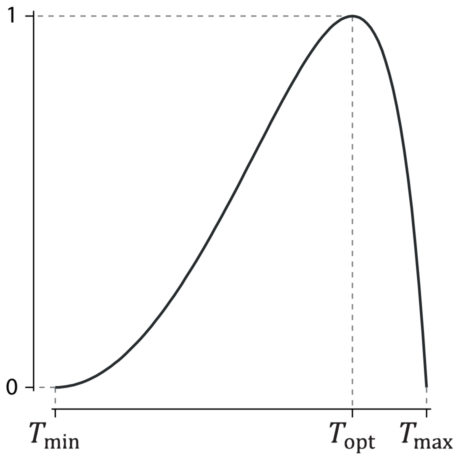

Figure S5: Plot of the Cardinal model function $g(T)$.

5. Dry mass measurements

Figure S6 explains which initial bacteria in Fig. 3 have been chosen over the field of view to plot the dry mass as a function of the temperature in Fig. 4.

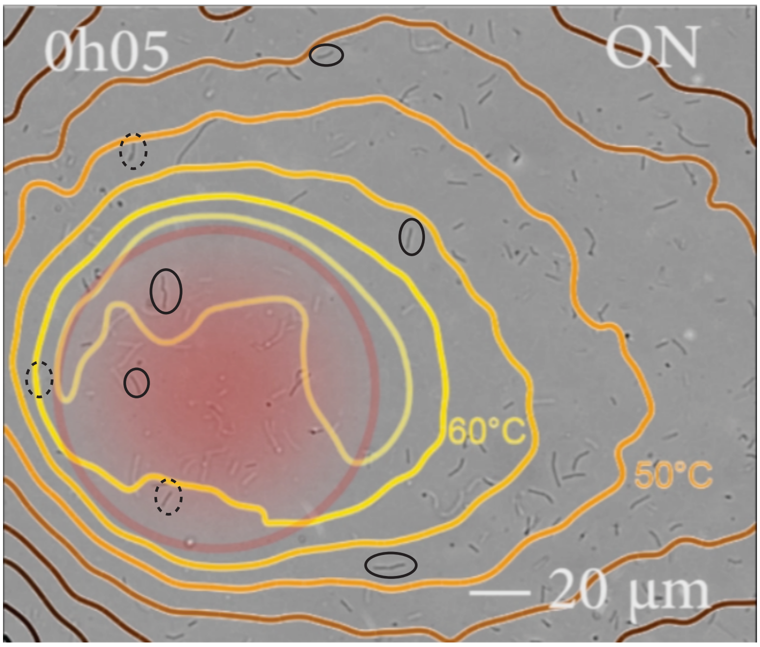

Figure S6: Image extracted from beginning of Movie M3. The 5 bacteria used for the plots of Figure 4a,b are encircled in black. Figure 4c also displays measurements of 3 other bacteria, which are indicated by dashed circles.

To illustrate the variability of the growth rate measurements from one experiment to another, especially in G_0, another dataset is presented hereinafter, related to Movie M4. Figure S7 displays an OT image of G. Stearothermophilus within a temperature gradient, where 6 bacteria are spotted.

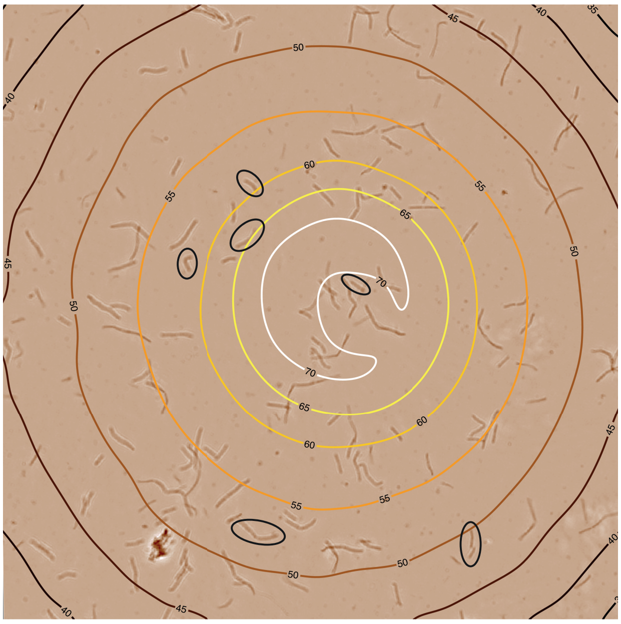

Figure S7: Image extracted from beginning of Movie M4. The 6 bacteria used for the plots of Figure S8a,b are encircled in black.

The dry mass of these bacteria were followed over time to build a growth rate curve (Fig. S8), just like with Figure 4. The fitting parameters are $(G_0;\ T_{min}; T_{opt}; T_{max}) = (0.49 \pm 0.06;\ 44 \pm 4;\ 64.6 \pm 0.7;\ 66 \pm 3)$ °C, to be compared with the fitting parameters of Figure 4: with $(G_0;\ T_{min}; T_{opt}; T_{max}) = (0.70 \pm 0.2;\ 40 \pm 4;\ 65 \pm 1.6;\ 67 \pm 3)$ °C

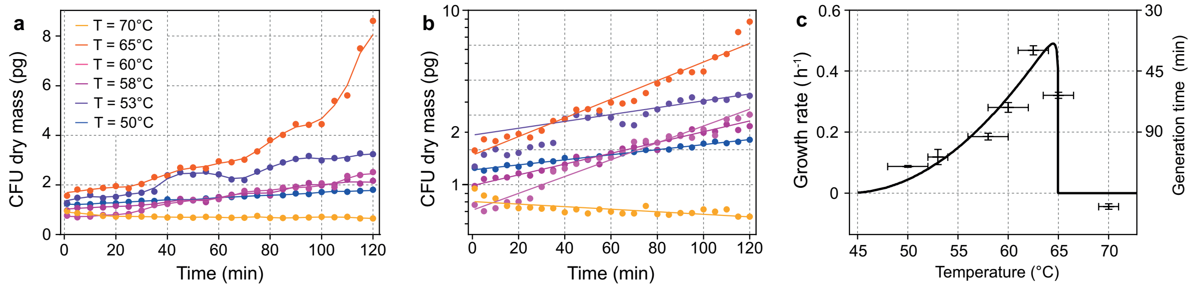

Figure S8: (a) Microbial growth at different temperatures. Data acquired from a single movie of bacteria growing within a temperature gradient (movie M4). (b) Same as (a) in a semi-log scale. (c) Growth rate τ and generation time g calculated from the linear fits of (b). Horizontal error bar: temperature range over which the mCFU expanded over the field of view during growth. Vertical error bar: root mean square errors of the linear regressions in (b).

Then, Figure S9 plots together the growth rates as a function of the temperature for the two data sets corresponding to Movies M3 and M4 (Figures 4c and S8c), demonstrating the typical variation of G_0 that can be observed.

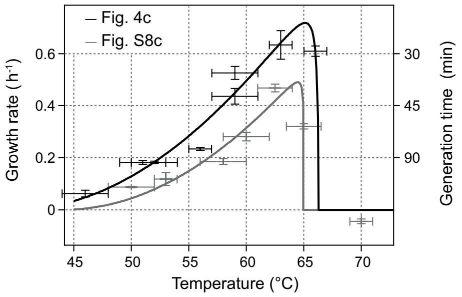

Figure S9: Comparison of the two growth rate functions plotted in Figs. 4c and S8c. Horizontal error bar: temperature range over which the mCFU expanded over the field of view during growth. Vertical error bar: root mean square errors of the linear regressions in (b).

6. Movies

Movie M1: `M1_GS_OnOff.mov`. Growth of *G. stearothermophilus* with the heating laser turned on, off and on, with the superimposition of the measured isotherms. The laser beam intensity is represented as well, in red.

Movie M2: `M2_GS_PreIncubation.mov`. Two videos displaying the growth of *G. stearothermophilus* through laser heating with (left side) and without (right side) pre-incubation of the sample. The laser beam, light disk, is depicted as a red disk when the laser is turned on (at $t = 5$ min). With pre-incubation, the growth starts a few minutes after the laser is turned on. The lag time is longer when using samples without pre-incubation (around 40 min).

Movie M3: `M3_GS_Growth_Fig2-4.mov`. Growth of *G. stearothermophilus* with the super-imposition of the measured isotherms. The laser beam intensity is represented as well, in red. It is shaped by the SLM to produce a uniform temperature distribution. Corresponds to the data presented in Figures 2, 3, 4.

Movie M4: `M4_GS_Growth_FigS7-9.avi`. Growth of *G. stearothermophilus* with the super-imposition of the measured isotherms. The laser beam is shaped by the SLM to produce a uniform temperature distribution. Corresponds to the data presented in Figures S7, S8, S9.

Movie M5: `M5_GS_Swim_Fig5.mov`. Swimming of *G. stearothermophilus* bacteria (real time). Some bacteria swim over the field of view as soon as the laser is turned on. The laser beam profile was uniform (not shaped by an SLM), producing a Gaussian-like temperature

distribution. Isotherms have been superimposed. The laser beam intensity is represented as well, in red. Corresponds to Figure 5.

Movie M6: `M6_GS_Germination_Fig6.avi`. Germination of G. stearothermophilus followed by cross-grating phase microscopy (CGM), and activated by laser-heating. Corresponds to Figure 6.

Movie M7: `M7_SS_Growth_Fig7.avi`. Growth of S. shibatae archaea followed by cross-grating phase microscopy (CGM), and activated by laser-heating. Corresponds to Figure 7.

7. References

1. Molinaro, C. *et al.* Are bacteria claustrophobic? The problem of micrometric spatial confinement for the culturing of micro-organisms. *RSC Adv.* **11**, 12500–12506 (2021).

2. Kakagianni, M., Gougouli, M. & Koutsoumanis, K. P. Development and application of Geobacillus stearothermophilus growth model for predicting spoilage of evaporated milk. *Food Microbiol.* **57**, 28–35 (2016).
