# Supplementary material for: Life at high temperature observed in vitro upon laser heating of gold nanoparticles
Source: Nat Commun. 2022 Sep 12;13:5342. doi: 10.1038/s41467-022-33074-6 (PMC9468142; doi:10.1038/s41467-022-33074-6)
Supplement: Supplementary file 3 — Description to Additional Supplementary Information [file 41467_2022_33074_MOESM3_ESM.pdf]

Description of Additional Supplementary Files

Legends for Movies

Movie M1: M1_GS_OnOff.mov. Growth of G. stearothermophilus with the heating laser turned on, off and on, with the superimposition of the measured isotherms. The laser beam intensity is represented as well, in red.

Movie M2: M2_GS_PreIncubation.mov. Two videos displaying the growth of G. stearothermophilus through laser heating with (left side) and without (right side) preincubation of the sample. The laser beam, light disk, is depicted as a red disk when the laser is turned on (at � = 5 min). With pre-incubation, the growth starts a few minutes after the laser is turned on. The lag time is longer when using samples without pre-incubation (around 40 min).

Movie M3: M3_GS_Growth_Fig2-4.mov. Growth of G. stearothermophilus with the superimposition of the measured isotherms. The laser beam intensity is represented as well, in red. It is shaped by the SLM to produce a uniform temperature distribution. Corresponds to the data presented in Figures 2, 3, 4.

Movie M4: M4_GS_Growth_FigS7-9.avi. Growth of G. stearothermophilus with the super-imposition of the measured isotherms. The laser beam is shaped by the SLM to produce a uniform temperature distribution. Corresponds to the data presented in Figures S7, S8, S9.

Movie M5: M5_GS_Swim_Fig5.mov. Swimming of G. stearothermophilus bacteria (real time). Some bacteria swim over the field of view as soon as the laser is turned on. The laser beam profile was uniform (not shaped by an SLM), producing a Gaussian-like temperature distribution. Isotherms have been superimposed. The laser beam intensity is represented as well, in red. Corresponds to Figure 5.

Movie M6: M6_GS_Germination_Fig6.avi. Germination of G. stearothermophilus

followed by cross-grating phase microscopy (CGM), and activated by laser-heating.

Corresponds to Figure 6.

Movie M7: M7_SS_Growth_Fig7.avi. Growth of S. shibatae archaea followed by crossgrating phase microscopy (CGM), and activated by laser-heating. Corresponds to Figure 7.
